# Intraoperative hemidiaphragm electrical stimulation reduces oxidative stress and upregulates autophagy in surgery patients undergoing mechanical ventilation: exploratory study

**DOI:** 10.1186/s12967-016-1060-0

**Published:** 2016-10-26

**Authors:** Robert T. Mankowski, Shakeel Ahmed, Thomas Beaver, Marvin Dirain, Chul Han, Phillip Hess, Tomas Martin, Barbara K. Smith, Shinichi Someya, Christiaan Leeuwenburgh, A. Daniel Martin

**Affiliations:** 1 Department of Aging and Geriatric Research, University of Florida, P.O.Box. 100107, Gainesville, FL 32611 USA; 2 Department of Physical Therapy, University of Florida, Box 100154, UFHSC, Gainesville, FL 32610-0154 USA; 3 Division of Thoracic and Cardiovascular Surgery, Department of Surgery, University of Florida, Gainesville, FL USA

## Abstract

**Background:**

Mechanical ventilation (MV) during a cardio-thoracic surgery contributes to diaphragm muscle dysfunction that impairs weaning and can lead to the ventilator- induced diaphragm dysfunction. Especially, it is critical in older adults who have lower muscle reparative capacity following MV. Reports have shown that the intraoperative intermittent hemidiaphragm electrical stimulation can maintain and/or improve post-surgery diaphragm function. In particular, from a molecular point of view, intermittent ES may reduce oxidative stress and increase regulatory autophagy levels, and therefore improve diaphragm function in animal studies. We have recently shown in humans that intraoperative ES attenuates mitochondrial dysfunction and force decline in single diaphragm muscle fibers. The aim of this study was to investigate an effect of ES on oxidative stress, antioxidant status and autophagy biomarker levels in the human diaphragm during surgery.

**Methods:**

One phrenic nerve was simulated with an external cardiac pacer in operated older subjects (62.4 ± 12.9 years) (n = 8) during the surgery. The patients received 30 pulses per min every 30 min. The muscle biopsy was collected from both hemidiaphragms and frozen for further analyses. 4-hydroxynonenal (4-HNE), an oxidative stress marker, and autophagy marker levels (Beclin-1 and the ratio of microtubule-associated protein light chain 3, I and II-LC3 II/I) protein concentrations were detected by the western blot technique. Antioxidant enzymatic activity copper-zinc (CuZnSOD) and manganese (MnSOD) superoxide dismutase were analyzed.

**Results:**

Levels of lipid peroxidation (4-HNE) were significantly lower in the stimulated side (p < 0.05). The antioxidant enzyme activities (CuZnSOD and MnSOD) in the stimulated side of the diaphragm were not different than in the unstimulated side (p > 0.05). Additionally, the protein concentrations of Beclin-1 and the LC3 II/I ratio were higher in the stimulated side (p < 0.05).

**Conclusion:**

These results suggest that the intraoperative electrical stimulation decreases oxidative stress levels and upregulates autophagy levels in the stimulated hemidiaphragm. These results may contribute future studies and clinical applications on reducing post-operative diaphragm dysfunction.

## Background

Mechanical ventilation (MV) is a life-saving component of modern intensive care and surgery. However, diaphragmatic unloading induced by MV may result in deleterious changes on the cellular level even within the first few hours from the intubation [1]. Diaphragm unloading may contribute to diaphragm muscle dysfunction that impairs weaning and can lead to the ventilator- induced diaphragm dysfunction (VIDD) [2]. Studies have shown that the intermittent intraoperative hemidiaphragm electrical stimulation may maintain or improve post-surgery diaphragm function [3–6]. For example, recently, we were the first to report that intraoperative hemidiaphragm electrical stimulation improved state III (25 %) and state IV (42 %) mitochondrial respiration in comparison with the unstimulated hemidiaphragm in older adults who underwent cardio-thoracic surgeries [6]. Moreover, single fiber force was improved by 30 % in the stimulated side of the diaphragm muscle in the same group of patients [5]. These results warranted further biochemical analyses investigating molecular pathways of these beneficial effects of electrical stimulation on diaphragm function.

Animal studies showed that diaphragmatic inactivity promotes reactive oxygen species (ROS) formation and mitochondrial dysfunction [7, 8] and may contribute to VIDD [9]. Potentially, excessive production of reactive oxygen species may be caused by mechanical inactivity of the respiratory muscles during MV—a state of metabolic oversupply [9, 10]. On the other hand, MV induces oxidative stress that may also reduce mitochondrial oxygen phosphorylation and may lead to reduced overall energy supply to muscle and to cell apoptosis (showed in cultured human diaphragm muscle cells) [11]. However, these molecular pathways have not been well studied in humans undergoing MV.

It has also been shown that autophagy is an important process in maintaining cell homeostasis by disposing cytotoxic elements of malfunctioned cell components e.g. mitochondrial turnover—mitophagy and thereby reducing pathophysiological ROS formation [12]. Importantly, levels of autophagy diminish with age, which may be an additive factor to diaphragm mitochondrial dysfunction in post-operative weaning from MV in older adults [13].

We hypothesized that intermittent electrical stimulation may reduce deleterious oxidative stress levels and increase basal regulatory autophagy levels, which maybe one of the key factors to improve diaphragm function during cardio-thoracic surgeries in mechanically ventilated patients [5, 6].

## Methods

### Subjects

Eight patients (at low risk for postoperative complications and weaning difficulties) undergoing cardiothoracic surgery at Shands Hospital at the University of Florida were recruited into the study (Table 1). The phrenic nerve was selected by the surgeons’ convenience and stimulated with an external cardiac pacer (Medtronic 5388) with temporary cardiac pacing wire electrodes. Stimulation was conducted for 1 min (30 pulses per min, 1.5 min duration) as soon as the phrenic nerve and diaphragm were exposed and every 30 min thereafter. Exclusion criteria included: prior surgery to the heart, diaphragm, pleura or phrenic nerves resulting in anatomical changes that would complicate obtaining muscle samples or interfere with phrenic stimulation; neuromuscular or inflammatory muscle diseases; obstructive lung disease (FEV1.0 < 60 % of predicted); other lung disease (bronchiectasis, lung cancer, pulmonary hypertension, tuberculosis or pulmonary fibrosis etc.); NYHA Class IV heart failure; implanted cardiac pacemaker or defibrillators; use of immunosuppressants, corticosteroids or aminoglycoside antibiotics within 60 days of surgery and serum creatinine >1.6 mg/dl. The detailed procedure description has been published elsewhere [6]. Full thickness diaphragm samples (20–50 mg) were obtained 30 min following the last stimulation bout. Muscle biopsies from each hemidiaphragm were frozen immediately and later prepared for protein immunoblotting. The University of Florida IRB approved this study protocol, and all subjects gave their consent for participation.

**Table 1:** Patient demographics and surgery description

Pt #	Age	Sex	Height (cm)	Weight (kg)	MV start to biopsy (min)	Surgical procedure
1	52	F	162.6	65.5	241	Triple CABG
2	47	M	175.3	84.5	209	Aortic valve replacement
3	68	M	167	90.1	331	Triple CABG
4	53	M	182.9	95.5	316	Ascending aortic aneurysm
5	56	F	175.1	90.0	329	Aortic graft, aortic valve replacement, CABG
6	79	M	167.6	81.8	334	Aortic valve replacement, double CABG
7	48	M	167.6	81.8	295	Triple CABG
8	72	F	165.1	106.8	209	Aortic valve replacement
Mean	62.4 ± 12.9		172.6 ± 6.7	92.5 ± 10.6	284.1 ± 53.8	

Data presented as mean ± SD

*CABG* coronary bypass graft

### Western blot

Equal amounts of protein (50 lg/condition) were resolved by 12 % sodium dodecyl sulfate (SDS)–gel electrophoresis and transferred to polyvinylidene difluoride (PVDF) membranes, according to a partially modified conventional protocol [14]. The immunodetection included the transfer (20 V for 25 min, per each membrane) and blocking of the membrane with western blot (WB) blocking solution (2 h at room temperature). After washing the membranes two times with TBST 1, the blots were incubated with the corresponding primary antibodies (Cell Signaling, USA) against 4-Hydroxynonenal (4-HNE), Peroxysome proliferator-activated receptor gamma coactivator 1-alpha (PGC-1α), Beclin-1, microtubule-associated protein 1 light chain 3 I and II (LC3) (1:000) and (incubated at 4 °C overnight. The LC3-II/I ratio was calculated based on densitometry analysis of both bands. The ratio is a widely used indicator of autophagy flux [15]. The membranes were washed two times with TBST 1 and subsequently incubated with their respective HRP-conjugated secondary antibodies (1:10,000) (1 h at room temperature). The detection of bound antibodies was visualized by chemiluminescence with enhanced chemiluminescence (ECL) substrate. Finally, a quantification analysis was performed with Image J software (NIH), using Ponceau stain.

### Antioxidant enzymatic activity

Superoxide dismutase (SOD) activity was measured as previously described [16].

## Data analysis

Two-tailed t-tests for matched pairs were used to compare distributions and statistical significance was set at p < 0.05. Data are shown as mean ± standard deviation.

## Results

### Stimulation

Stimulation of a hemidiaphragm was well tolerated and the biopsies were obtained without complication. All subjects were extubated between 6.7 and 67.1 h after surgery (27.5 ± 22.8 h). The first stimulation was initialized when the phrenic nerve and diaphragm were visualized by a surgeon (95.9 ± 12 min after intubation). The patients received an average of 6.4 ± 1.8 stimulation bouts with the mean amplitude of 19.6 ± 5.8 mA. Muscle biopsies were harvested 28.3 ± 1.8 min after the last stimulation bout.

### Biopsy analysis

**Fig. 1:**
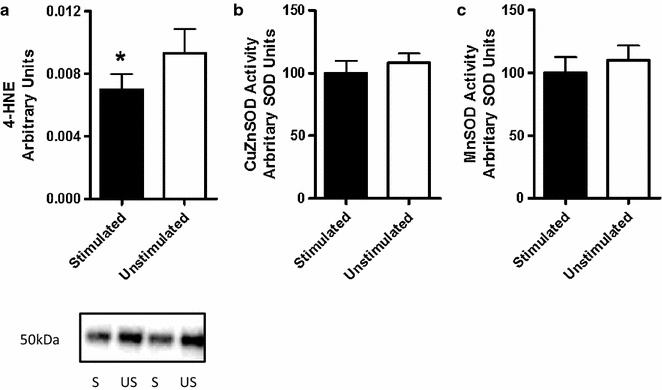
Levels of oxidative stress (**a**) and enzymatic activity (**b**, **c**) in the unstimulated and stimulated hemidiaphragm

**Fig. 2:**
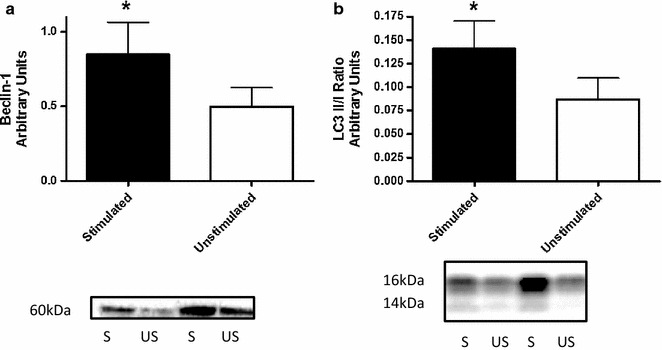
Protein concentrations of autophagy proteins (**a** Beclin-1 and **b** LC3-II/I ratio) in the unstimulated and stimulated diaphragm sides

Due to the limited sample availability we were only able to measure 4-HNE, Beclin-1, LC3 and PGC-1α protein concentrations and enzyme activity of CuZnSOD and MnSOD. Protein concentration of 4-HNE, a marker of lipid peroxidation levels and oxidative stress was significantly lower (p < 0.05) in the stimulated hemidiaphragm. Antioxidant enzyme activities of CuZnSOD and MnSOD were not different (p > 0.05) between the hemidiaphragms (Fig. 1). Macroautophagy biomarker levels of Beclin-1 and the LC3II/I ratio were significantly higher in the stimulated side (p < 0.05) (Fig. 2). Additionally, there was no difference in protein concentration levels of master regulator of mitochondrial biogenesis—PGC-1α between the hemidiaphragms.

## Discussion

We are the first to report potentially beneficial effects of electrical stimulation on oxidative stress and autophagy levels in human diaphragm in mechanically ventilated older patients during cardio-thoracic surgeries. The main finding of this exploratory study was that levels of oxidative stress were lower in the stimulated hemidiaphragm. Additionally, higher levels of autophagy levels markers i.e. Beclin-1 and the LC3-II/I ratio in the stimulated hemidiaphragm suggest upregulation of macroautophagy levels.

Previous reports suggested that higher levels of oxidative stress may be a key factor of the majority of pathways leading to VIDD in the mechanically ventilated patients, with mitochondrial dysfunction being an essential part [11, 17]. We reported that electrical stimulation improved mitochondrial function (i.e. state III and IV mitochondrial respiration) in comparison with the unstimulated hemidiaphragm [6]. These results suggest improved mitochondrial function in the stimulated hemidiaphragm, which maybe a contributing factor to reducing risk of VIDD in surgery patients. Mitochondrial function can be improved by generating new mitochondrial and/or removing the dysfunctional mitochondria (mitophagy) that are the source of oxidative stress [18]. In the current analysis we showed no difference in the protein concentration of PGC-1α, a biomarker of mitochondrial biogenesis levels. This suggests that autophagy-related mechanisms and reduced oxidative stress levels may have improved mitochondrial function in the stimulated hemidiaphragm [19] and that our observation is not due to an altered level of mitochondrial biogenesis.

The link between increased oxidative stress and MV-related diaphragm dysfunction has also been shown in animal models [17, 20]. Indeed, we found increases in 4-HNE protein concentration, a biomarker of lipid peroxidation, and thus an indication of increased oxidative stress levels in the unstimulated hemidiaphragm. Additionally, the activity levels of endogenous antioxidant defense system (CuZnSOD and MnSOD) were not different between the hemidiaphragms that may be associated with a limited samples size. The future studies may include measurements of other biomarkers of oxidative stress levels e.g. protein carbonylation and 8-isoprostane levels to better interpret the increased 4-HNE levels in the current analysis.

**Fig. 3:**
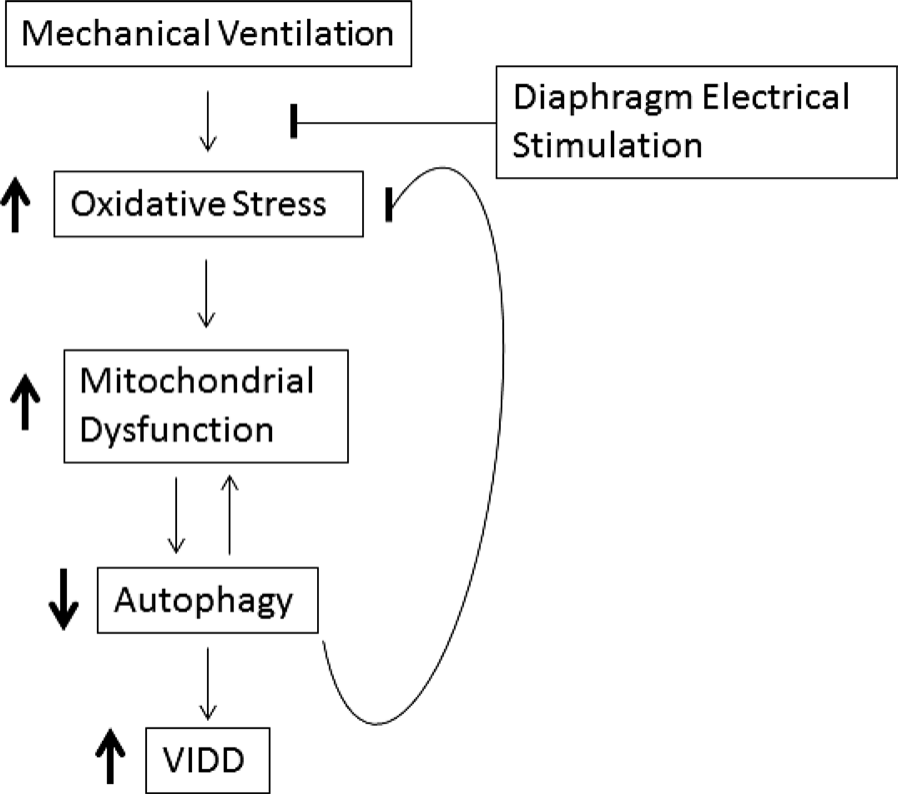
Conceptual figure of a potential mechanism of electrical stimulation and improved diaphragm function

Moreover, high levels of oxidative stress and autophagy were linked to apoptotic and proteolytic processes that had consequences in diaphragm dysfunction and developed VIDD in mechanically ventilated patients [2, 21]. However, recent reports suggest that *physiological* levels of ROS formation and autophagy are essential for cellular signaling and homeostasis [22]. In particular, ROS generated by the mitochondrial respiratory chain at low level stimulate cell signaling, but higher levels of ROS lead to mitochondrial damage that produce more oxidative stress [20]. Therefore, autophagy is suggested to play an important role in down-regulation of oxidative stress [19]. In particular, previous reports have shown that autophagy is essential in maintaining mitochondrial function (mitophagy) by degrading malfunctioning mitochondria that are one of the main sources of deleterious oxidative stress [13, 23]. However, other studies reported a link between higher levels of autophagy and human diaphragm atrophy in mechanically ventilated patients [21]. For examples, Hussain et al. reported higher levels of oxidative stress (4-HNE expression) and upregulated autophagy levels (LC3-II/I ratio) and evidence of autophagosome formation in chronically ventilated patients (average MV time 59 ± 16.5 h) in comparison with a control group (2–4 h of MV) [2, 21]. Also, animal studies have shown that diaphragm function preservation was accompanied by lower levels of oxidative stress and autophagy [20]. However, formation of ROS upregulates autophagy to remove defective proteins and malfunctioning mitochondria that would generate uncontrolled ROS and increase oxidative stress levels [19, 24, 25]. Accordingly, our results demonstrated lower levels of oxidative stress and higher levels of autophagy biomarkers, Beclin-1 and the LC3-II/I ratio, that suggest increased autophagosome formation and expansion levels (Fig. 2). Upregulation of Beclin-1 expression is needed for appropriate autophagy induction phase [22]. Although, the LC3-II/I ratio was higher in the stimulated side, the concentrations of LC3II were lower than LC3I in both hemidiaphragms, which suggests low levels of autophagic flux. For more accurate measurements of autophagy levels in order to answer a question if electrical stimulation promotes an autophagy-induced reduction of oxidative stress in the diaphragm, the future studies will involve electron microscopy documentation of autophagosomes in the surgery patient [26]. Additionally, it has been shown that 4-HNE induced an increase of autophagy levels by accumulation of LC3-II in vascular smooth cells [27, 28]. These results suggest a potential link between ROS-induced autophagy leading to autophagy-induced cellular homeostasis and lower oxidative stress levels as a result of autophagy-related cell survival during oxidative stress in the stimulated hemidiaphragm in surgery patients (Fig. 3).

Taken together, we can speculate that decreased oxidative stress levels maybe a product of increased cell-protective autophagy levels (Fig. 3). Although, these results may support our previous report of improved mitochondrial function in the stimulated hemidiaphragms, these exploratory results warrant future studies involving confocal microscopy methods in order to study the autophagy flux and support the clinical value of the diaphragm electrical stimulation.

## Study limitations

Although these results suggest ES has a potential protective effect against VIDD, this study has a few limitations. A larger sample size would increase the statistical power and the ability to detect more differences between stimulated and control tissue. Additionally, studying the biological pathways more comprehensively would have been informative, but was limited by small muscle specimens (~20 mg). However, despite the relatively small number of subjects and dependent variables examined, this study presents novel and important findings regarding the effect of electrical stimulation on the human diaphragm during surgery and MV.

## Conclusion

Intraoperative hemidiaphragm electrical stimulation may decrease oxidative damage and upregulate autophagy levels. Further studies on molecular bases of the diaphragm electrical stimulation are warranted on a larger population and using confocal technologies. These results may contribute future studies and clinical applications on reducing post-operative diaphragm dysfunction.

## Abbreviations

MV: mechanical ventilation
VIDD: ventilator induced diaphragm dysfunction
NYHA: New York Heart Association
FEV-1: forced expiratory volume 1
ROS: reactive oxygen species
IRB: Institutional Review Board
SDS: sodium dodecyl sulfate
PVDF: polyvinylidene difluoride
WB: western blot
4-HNE: 4-Hydroxynonenal
ECL: enhanced chemiluminescence
LC3: microtubule-associated protein light chain 3
CuZnSOD: copper-zinc superoxide dismutase
MnSOD: manganese superoxide dismutase
